# Molecular Characterization and Genetic Diversity of *Ehrlichia canis* in Naturally Infected Dogs from the Unaí Microregion, Minas Gerais, Brazil

**DOI:** 10.1007/s11686-026-01389-9

**Published:** 2026-09-07

**Authors:** Aroldo de Souza Junior, Jônathan David Ribas Chagas, Isadora dos Santos Dias, Ellen Meireles Brandão, Luiza Sena Chalub, Laura Marques Batista, Julia Oliveira Amaral, Jenevaldo Barbosa da Silva, Matheus Dias Cordeiro, Claudia Bezerra da Silva, Bruna de Azevedo Baêta

**Affiliations:** 1 Department of Animal Parasitology, Veterinary Institute, Federal Rural University of Rio de Janeiro, BR465-km07, Seropédica, Rio de Janeiro 23890-000 Brazil; 2 Institute of Agricultural Sciences, Federal University of the Jequitinhonha and Mucuri Valleys, Avenida Universitária nº1000, Unaí, Minas Gerais 38610-000 Brazil; 3 Department of Epidemiology and Public Health, Veterinary Institute, Federal Rural University of Rio de Janeiro, BR465-km07, Seropédica, Rio de Janeiro 23890-000 Brazil

**Keywords:** Genetic diversity, *Tandem repeats*, *trp19*, *p28*, *trp36*

## Abstract

*Ehrlichia canis* is an obligate intracellular bacterium responsible for canine monocytic ehrlichiosis and is widely distributed in tropical and subtropical regions. This study investigated the occurrence and genetic diversity of *E. canis* in naturally infected dogs from the Unaí microregion, Minas Gerais, Brazil, using the *trp19*, *p28*, and *trp36* genes as molecular markers. A total of 386 whole blood samples were initially screened by conventional PCR targeting the Anaplasmataceae 16S rRNA gene, yielding 38 positive samples (9.8%). Subsequent PCR targeting the *E. canis trp19* gene confirmed infection in 21 samples (5.4%). Among these, 12 samples were positive for the p28 gene and eight for the *trp36* gene. Sequence analysis of *p28* revealed high nucleotide identity (99.27–99.88%) with reference strains and amino acid substitutions. Haplotype network analysis identified nine *p28* haplotypes, indicating substantial intraspecific genetic diversity. Analysis of the *trp36* gene demonstrated the co-circulation of Brazilian and American genogroups, with variation in tandem repeat copy number. These findings reveal a genetically heterogeneous *E. canis* population in the Unaí microregion and provide evidence of the co-circulation of distinct lineages. The combined use of multiple molecular markers improved the characterization of the circulating strains and contributes to a better understanding of the molecular epidemiology and population diversity of *E. canis* in Brazil.

## Introduction

Tick-borne diseases represent a major concern in veterinary medicine due to their impact on the health and productivity of domestic and production animals, which are frequently exposed to tick vectors carrying a wide range of infectious agents [1]. Among these pathogens, bacteria of the genus *Ehrlichia*, particularly *Ehrlichia canis*, are of major importance due to their high prevalence in dogs and the clinical severity of canine monocytic ehrlichiosis (CME) [2].

*Ehrlichia canis* is a Gram-negative, obligate intracellular bacterium of the family Anaplasmataceae that parasitizes cells of the mononuclear phagocytic system [3]. It is widely distributed in tropical and subtropical regions, where its transmission occurs mainly through the bite of infected ixodid ticks, particularly *Rhipicephalus linnaei* during blood feeding [4]. The clinical manifestations of CME vary according to the stage of infection (acute, subclinical, or chronic), ranging from nonspecific systemic signs to severe hematological alterations such as anemia, hemorrhages, and weight loss [5–6].

From a molecular perspective, *E. canis* exhibits limited variability in conserved genes such as 16S rRNA and *dsb*, which has led to the use of more variable loci for genetic characterization. Among these, the outer membrane protein gene *p28*, encoded by a multigene family with multiple allelic variants, and the immunoreactive glycoprotein gene *trp36* have been widely used for strain differentiation and genogroup identification [7–12].

Despite the recognized importance of canine monocytic ehrlichiosis in Brazil, molecular data regarding the genetic structure of *E. canis* in several regions remain scarce, particularly in the Northwest of Minas Gerais. Furthermore, little is known about the co-circulation of distinct genogroups and the population structure of the agent in naturally infected canine populations in this region. Understanding the genetic diversity and distribution of *E. canis* is essential to elucidate its epidemiological dynamics and evolutionary patterns in endemic areas.

In this context, the present study aimed to detect and molecularly characterize *E. canis* from naturally infected dogs in the Unaí microregion, Northwest Minas Gerais, Brazil, using the *p28* and *trp36* genes as molecular markers to assess genetic diversity and infer population structure.

## Materials and Methods

### Study Area, Animal Selection, and Sample Collection

The present study was conducted using animals from the Unaí microregion, located in the Northwest Mesoregion of Minas Gerais (MG), Brazil. The microregion covers a territorial area of 27,384 km^2^ and has a predominantly tropical climate, comprising the municipalities of Arinos, Bonfinópolis de Minas, Buritis, Cabeceira Grande, Dom Bosco, Formoso, Natalândia, Unaí, and Uruana de Minas.

The minimum sample size required for molecular analyses was calculated using the formula described by Agranonik and Hirakata [13], which considers the confidence level, the estimated proportion, and the acceptable margin of error. The formula is expressed as follows: n = (Z^2^ × P × (1 − P) / e^2^), where n is the sample size, Z is the critical value of the normal distribution for a 95% confidence level (1.96), P is the estimated population proportion (in this case defined as 50%), and e is the margin of error (5%). Substituting these values into the equation resulted in a minimum sample size of 384 individuals.

For blood sample collection, 3 mL of blood was obtained by cephalic venipuncture with the owners’ consent. The samples were placed in tubes containing the anticoagulant ethylenediaminetetraacetic acid (EDTA) and subsequently stored under refrigeration. Initially, the samples were processed at the Laboratory of Parasitic Diseases of the Institute of Agricultural Sciences of the Federal University of the Jequitinhonha and Mucuri Valleys (ICA/UFVJM), where a screening step was performed. The material was then sent to the Laboratory of Study of Parasite–Host Interaction (LEIPH), at the Federal Rural University of Rio de Janeiro (UFRRJ), for molecular analyses. A total of 386 dogs of both sexes, with no restrictions regarding breed or age, were included in the study. Dogs were sampled from veterinary clinics and/or rural settlements in the Unaí microregion, Minas Gerais, Brazil, using convenience sampling. The inclusion criterion was the availability of dogs for blood collection during the sampling period.

### DNA Extraction and Endogenous Gene Verification

Genomic DNA extraction was performed from 300 µL of whole blood using the commercial Wizard^®^ Genomic DNA Purification Kit (Promega^®^), according to the manufacturer’s instructions. After extraction, the samples were placed in properly labeled polypropylene microtubes and stored at − 20 °C until molecular analyses were performed.

The presence of amplifiable DNA was confirmed by conventional PCR targeting the glyceraldehyde-3-phosphate dehydrogenase (GAPDH) enzyme, amplifying a fragment of approximately 400 base pairs (bp). For this reaction, the primers GAPDH-F (5′-CCTTCATTGACCTCAACTACAT-3′) and GAPDH-R (5′-CCAAAGTTGTCATGGATGACC-3′) were used, as described by Birkenheuer, Levy, and Breitschwerdt [14].

PCR reactions were performed in a final volume of 25 µL, containing 2 µL of DNA template. The reaction mixture had the following final concentrations: 1 U of Taq DNA polymerase (GoTaq^®^, Promega^®^), 0.8 µM of each primer, 0.2 mM of each dNTP, 1.5 mM MgCl_2_, and 1X reaction buffer (Colorless GoTaq^®^, Promega^®^). The final volume was completed with DNase-Free ultrapure water. DNase-Free ultrapure water was used as a negative control in place of template DNA.

Amplification was carried out in a T100™ thermocycler (Bio-Rad Laboratories Brazil^®^) under the following cycling conditions: initial denaturation at 95 °C for 2 min and 30 s, followed by 35 cycles of denaturation at 95 °C for 30 s, annealing at 55 °C for 30 s, and extension at 72 °C for 25 s, with a final extension at 72 °C for 5 min. After confirmation of *gapdh* gene amplification, the samples were subjected to specific PCR assays for the target genes of interest.

### Detection of Anaplasmataceae Agents 16S rRNA Gene and *E. canistrp19* Gene

Conventional PCR screening was performed using the primers 750F (5′-TAGTCCACGCTGTAAACG-3′) and EC3 (5′-ACCCTAGTCACTAACCCAAC-3′) [15], targeting an approximately 691 bp fragment of the 16S rRNA gene for the detection of members of the family Anaplasmataceae, in a final reaction volume of 25 µL.

The reactions were carried out using 1X buffer (Colorless GoTaq^®^, Promega^®^), 2.0 mM MgCl₂ (Promega^®^ MgCl₂ Solution), 0.2 mM dNTPs, 0.8 µM of each primer, and 1 U of Taq DNA polymerase (GoTaq^®^, Promega^®^). Thermocycling conditions consisted of an initial denaturation at 95 °C for 5 min, followed by 40 cycles of denaturation at 95 °C for 15 s, annealing at 55 °C for 15 s, and extension at 72 °C for 30 s, with a final extension at 72 °C for 5 min.

Samples that tested positive in the Anaplasmataceae 16S rRNA PCR were subsequently subjected to a PCR assay targeting the *trp19* gene for the detection of *E. canis*. This PCR amplified a 414 bp fragment. The primers used were EC19-F1 (5′-ATTAGTGTTGTGGTTATGCAA-3′) and EC19-R1 (5′-TACGCTTGCTGAATATCATGA-3′) [16].

The reactions were carried out using 1X buffer (Colorless GoTaq^®^, Promega^®^), 2.5 mM MgCl₂ (Promega^®^ MgCl₂ Solution), 0.2 mM dNTPs, 0.6 µM of each primer, and 1 U of Taq DNA polymerase (GoTaq^®^, Promega^®^).

Amplification conditions consisted of an initial denaturation at 94 °C for 3 min, followed by 35 cycles of denaturation at 94 °C for 1 min, annealing at 55 °C for 1 min, and extension at 72 °C for 1.5 min. A final extension step was performed at 72 °C for 5 min.

### Molecular Characterization of *E. canis* Using the *p28* and *trp36* Genes

For molecular characterization, the samples were subjected to amplification of a fragment of the *p28* gene, aiming at obtaining a product of approximately 843 base pairs (bp), using the primers ECp28-F (5'-ATGAATTGCAAAAAAATTCTTATA-3') and ECp28-R (5'-TTAGAAGTTAAATCTTCCTCC-3') [17].

PCR reactions were performed in a final volume of 25 µL, containing 2 µL of extracted DNA, 1X reaction buffer (Colorless GoTaq^®^, Promega^®^), 2.5 mM MgCl₂ (Promega^®^ MgCl₂ Solution), 0.2 mM of each dNTP, 0.5 µM of each primer, and 1 U of Taq DNA polymerase (GoTaq^®^, Promega^®^). The final volume was completed with DNase-Free ultrapure water.

Amplification was carried out in a thermocycler under the following conditions: initial denaturation at 95 °C for 2 min and 30 s, followed by 40 cycles of denaturation at 95 °C for 30 s, annealing at 55 °C for 30 s, and extension at 72 °C for 50 s, with a final extension step at 72 °C for 5 min.

For amplification of a fragment of the *trp36* gene, samples previously positive for *E. canis* by PCR targeting the *p28* gene were subjected to a semi-nested PCR. In the first reaction, the primers F: 5′-TTTAAAACAAAATTAACACACTA-3’ and R: 5′-AAGATTAACTTAATACTCAATATTACT-3′ were used, and in the second reaction the primer F: 5′-CACACTAAAATGTATAATAAAGC-3′ [18]. The first reaction was performed in a final volume of 25 µL containing 3 µL of DNA, 1X reaction buffer (Colorless GoTaq^®^, Promega^®^), 1.5 mM MgCl₂, 0.2 mM of each dNTP, 0.8 µM of each primer, and 1 U of Taq DNA polymerase (GoTaq^®^, Promega^®^), with the final volume completed with DNase-Free ultrapure water. For the second reaction, the same conditions were maintained, using 2 µL of the first PCR product as DNA template.

Thermocycling conditions for both steps of the semi-nested PCR consisted of initial denaturation at 95 °C for 3 min, followed by 35 cycles of denaturation at 95 °C for 30 s, annealing at 54 °C for 30 s, and extension at 72 °C for 1 min, with a final extension at 72 °C for 5 min.

As a positive control, *E. canis* DNA obtained from naturally infected canine blood, confirmed by genetic sequencing [4], was used. DNase-Free ultrapure water was used as a negative control in all reactions.

### Electrophoresis and Analysis of Results

The amplified products were separated on 1.5% agarose gel (UltraPureTM LMP Agarose, Invitrogen^®^) under a current of 75 V (5 V/cm) for 45 min in TAE (Tris-acetate-EDTA). Subsequently, they were stained with ethidium bromide (0.5 µg/mL) and visualized using a UV transilluminator (L-PIX Sti, Loccus). A 1 kb DNA Ladder (Promega^®^) molecular weight marker was used to determine fragment sizes.

### Purification and Genetic Sequencing

PCR products from positive samples for the *p28* and *trp36* genes were purified from 5 µL aliquots using ExoSAP-IT (Thermo Fisher Scientific), according to the manufacturer’s recommendations. All sequencing reactions were performed in both directions in an Applied Biosystems^®^ 9700 thermocycler, and the products were sequenced using an Applied Biosystems^®^ genetic analyzer, model 3500.

### Haplotype Analysis

Haplotype analysis was conducted to investigate the population structure and evolutionary dynamics of *E. canis* strains circulating among dogs in the state of Minas Gerais. Initially, main polymorphism indices were calculated using DnaSP v6.12.03, including the number of polymorphic sites (S), total number of haplotypes (H), haplotype diversity (Hd), and nucleotide diversity (π) [19]. Estimates were obtained after removal of gaps and exclusion of invariant sites, ensuring that only informative positions contributed to genetic variability inference.

Then, to visualize haplotype distribution and frequency, a haplotype network was constructed using the median-joining method in PopART v1.7. The dataset included 11 *p28* gene sequences obtained in this study and 19 reference sequences from different locations, including Brazil, United States, India, Philippines, and Vietnam.

### Phylogenetic analysis

Chromatograms were analyzed and nucleotide sequences were assembled and edited using CLC Main Workbench 23 software (CLC Bio-Qiagen). Sequences were then subjected to similarity analysis using BLASTn against the NCBI GenBank nucleotide database. Previously published homologous sequences from other studies were retrieved in FASTA format and aligned with the sequences obtained in this study using the multiple alignment algorithm ClustalW implemented in MEGA X [20]. The aligned dataset was subsequently trimmed.

Phylogenetic analysis of *E. canis* was performed based on datasets comprising 792 bp of the *p28* gene with 30 *E. canis* sequences and 942 bp of the *trp36* gene with 28 sequences. Outgroup sequences used were *Ehrlichia chaffeensis* strain Arkansas (CP000236) for *p28* and *E. chaffeensis* (DQ085431) for the *trp36* gene.

Phylogenetic trees were inferred using the Maximum Likelihood (ML) method, applying the Tamura 3-parameter model with Gamma distribution (T92 + G) for both *p28* and *trp36* genes. Analyses were performed in MEGA X [20], with model selection based on the Akaike Information Criterion (AIC). Statistical support for clades was estimated by bootstrap analysis with 1,000 replicates using heuristic search. Final tree formatting was performed using Inkscape v1.3.2.

The datasets generated and analyzed during the present study are available in the NCBI GenBank database (https://www.ncbi.nlm.nih.gov/genbank/) and can be accessed through accession numbers PZ138103–PZ138113 for the *p28* gene and PZ138114–PZ138118 for the *trp36* gene.

## Results

All samples analyzed in the present study showed amplification of an approximately 400 bp fragment corresponding to the endogenous *gapdh* gene, confirming the quality and integrity of the extracted DNA.

Screening PCR targeting the Anaplasmataceae 16S rRNA gene detected 38 positive samples among the 386 analyzed (9.8%). Of these, 21 were subsequently confirmed as *E. canis* by PCR amplification of the *trp19* gene, resulting in an overall positivity rate of 5.4% (21/386). Subsequently, the positive samples were analyzed by PCR targeting the *p28* gene, in which a 843 bp fragment was observed in 57.1% (12/21) of the samples evaluated. All samples positive for the *p28* gene were submitted to genetic sequencing; however, one sample showed a low-quality chromatogram, preventing the generation of a reliable sequence. Thus, 11 samples were included in the phylogenetic analyses.

When subjected to amplification of the *trp36* gene of *E. canis*, eight of the 21 samples previously positive for the agent showed amplification. After sequencing, five samples were included in the genetic diversity analyses.

In the analysis of nucleotide sequence identity obtained from the *p28* gene, the sequences generated in the present study showed identity ranging from 99.27 to 99.88% when compared to reference sequences of *E. canis* strain Jake (CP000107) and *E. canis* strain Jaboticabal (EF014897).

For the analysis of *p28* gene sequences, nucleotides were translated into amino acids in order to identify mutation sites. The sequences that showed the highest identity with *E. canis* strain Jake (CP000107) used this strain as a reference, whereas those that demonstrated higher identity with *E. canis* strain Jaboticabal (EF014897) had this latter adopted as the reference sequence for comparisons.

The comparison of amino acid sequences of the *p28* gene with the reference strain *E. canis* Jake demonstrated 11 mutation sites, located at positions 64, 68, 79, 80, 82, 142, 144, 150, 152, 203, and 253 (Table 1). Amino acid substitutions occurred variably among the analyzed samples, evidencing intraspecific polymorphism.

**Table 1:** Amino acid substitutions in the *p28* gene of *E. canis* compared to the reference sequence CP000107

Sequences	Amino acid position											
	64	68	79	80	82	142	144	150	152	203	253	
*E. canis* strain Jake (CP000107)	K	N	N	I	T	H	S	S	S	N	E	
Sample 8 (PZ138103)	.	.	S	.	K	.	.	.	D	.	K	
Sample 20 (PZ138105)	A	.	S	.	K	.	.	.	D	.	.	
Sample 21 (PZ138106)	R	D	.	T	.	.	P	.	.	.	.	
Sample 24 (PZ138107)	R	D	.	T	.	.	.	.	.	.	.	
Sample 38 (PZ138109)	R	D	.	T	.	.	.	.	.	.	.	
Sample 45 (PZ138111)	R	D	.	T	.	.	P	P	.	.	.	
Sample 49 (PZ138112)	.	.	.	.	.	.	.	.	.	S	.	
Sample 404 (PZ138113)	.	.	.	.	.	N	.	.	.	.	.	

*Position based on the *E. canis* sequence (CP000107). The dots “.” represent the conserved regions

The comparison of *p28* gene sequences with the *E. canis* strain Jaboticabal reference strain showed point amino acid substitutions at positions 81, 153, 154, 156, and 208 (Table 2), observed variably among the analyzed samples, which showed high similarity to the reference sequence.

**Table 2:** Amino acid substitutions in the *p28* gene of *E. canis* compared to the reference sequence EF014897

Sequences	Amino acid position				
	81	153	154	156	208
*E. canis* strain Jaboticabal (EF014897)	P	S	S	S	P
Sample 9 (PZ138104)	.	.	N	.	.
Sample 26 (PZ138108)	.	.	.	N	T
Sample 40 (PZ138110)	L	L	.	.	.

*Position based on the *E. canis* sequence (EF014897). The dots “.” represent the conserved regions

**Fig. 1:**
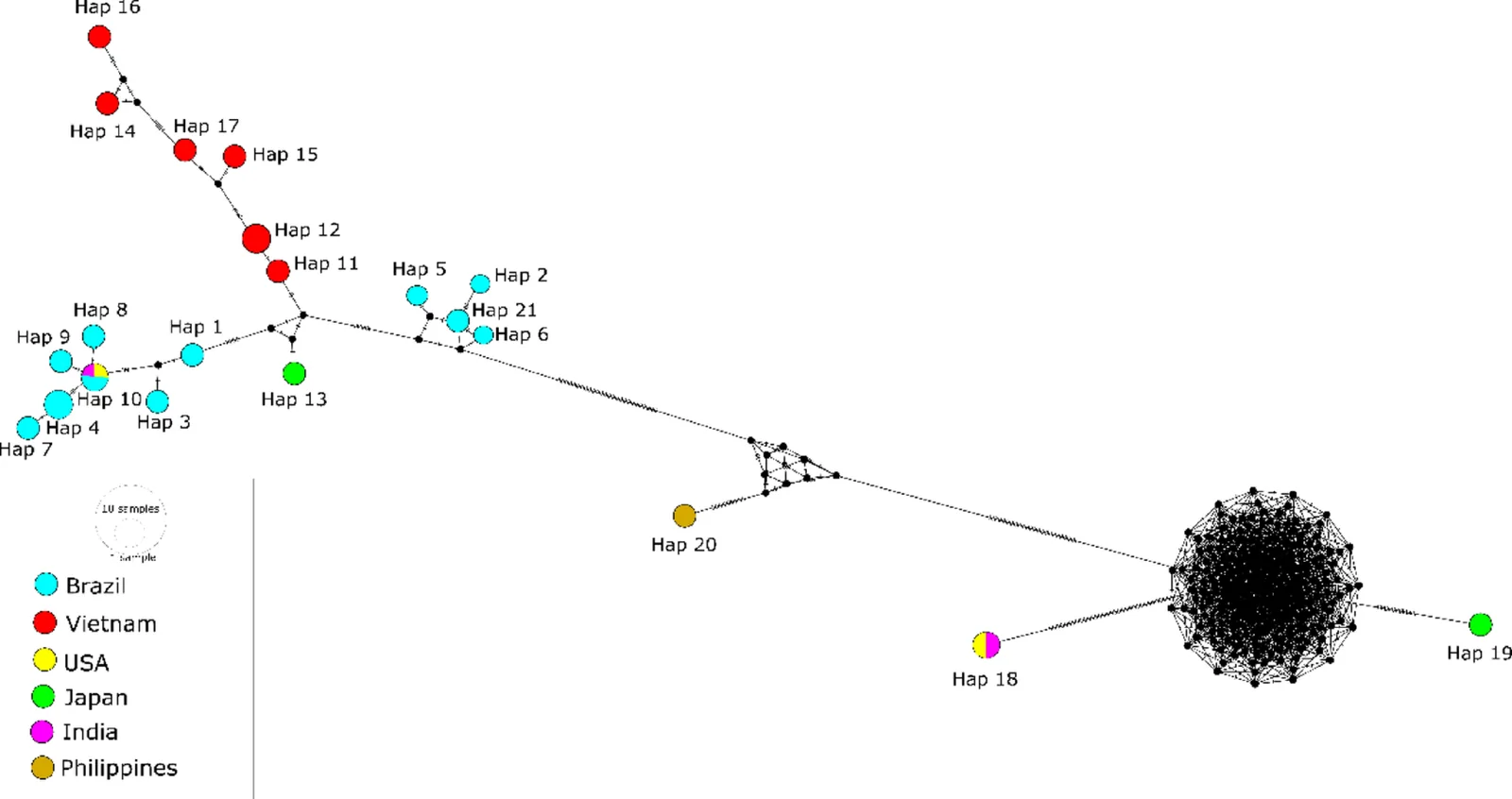
Median-joining haplotype network based on *p28* sequences of *E. canis* isolates detected in Unaí (MG), Brazil, and in foreign countries. Small differences between haplotypes represent mutational events. Black circles represent intermediate nodes generated by single nucleotide polymorphisms (SNPs)

Considering the global set of sequences, 21 distinct haplotypes were identified in the *p28* gene. Among these, nine haplotypes were exclusive to the samples obtained in the present study (Hap 1–9), with haplotype diversity (Hd) of 0.9632, nucleotide diversity (π) of 0.0731, and 224 polymorphic sites (S). The resulting network revealed a diverse scenario characterized by multiple haplotypes exclusive to the Unaí microregion, separated from exogenous sequences by numerous mutational steps. No haplotype was dominant, with the most frequent one (Hap 4) present in only three samples, evidencing a pattern of genetic dispersion (Fig. 1).

**Fig. 2:**
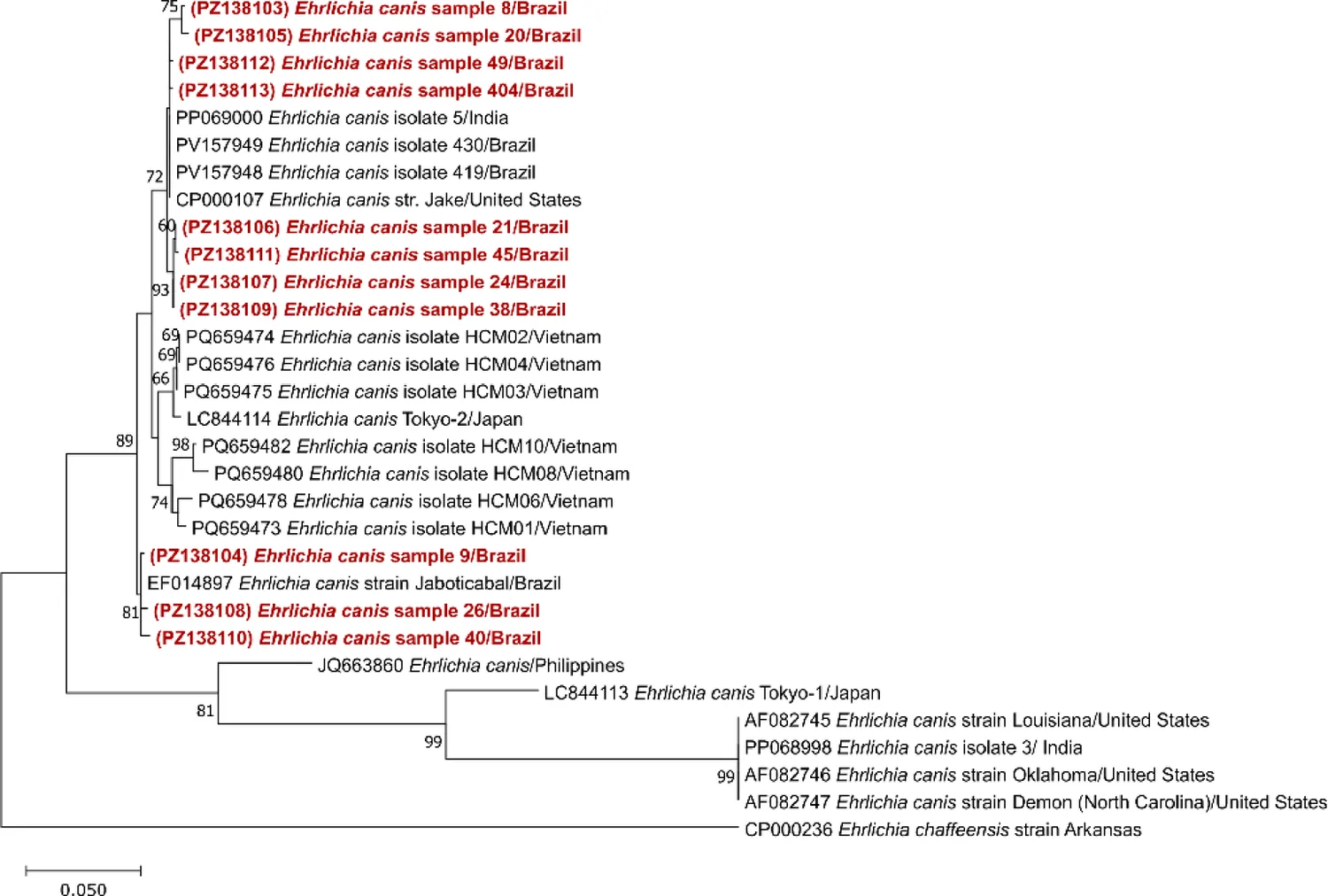
Phylogenetic tree based on a fragment of the *p28* gene from dogs in the Unaí microregion, MG, Brazil, constructed using the Maximum Likelihood method and the Tamura 3-parameter model. Bootstrap values are shown next to the branches. Among-site rate variation was modeled using a discrete Gamma distribution (+ G, parameter = 0.6816). The scale bar indicates the number of substitutions per site

In the clustering analysis of the *p28* gene, eight sequences from the study were grouped with *E. canis* sequences from Rio de Janeiro, Brazil (PV157949; PV15794), India (PP690000), and the United States (CP000107). In contrast, three samples clustered with *E. canis* strain Jaboticabal, Brazil (EF014897), evidencing intraspecific diversity among the sequences (Fig. 2).

In the nucleotide sequence identity analysis of the *trp36* gene, the sequences generated in the present study showed identity ranging from 97% to 99% when compared with *E. canis* sequences available in the GenBank database from different geographic regions (MN159544; MW229079; ON886370; JX312078; DQ085427).

For the analysis of the *trp36* gene sequences, nucleotides were translated into amino acids. Four sequences encoded nine amino acids (TEDSVSAPA) with the number of repeats varying from 12 to 16 copies, whereas one sequence encoded a tandem region composed of eight amino acids (ASVVPEAE) with 13 repeats (Table 3).

**Table 3:** *Tandem repeats* and copy number among *trp36* gene sequences of *E. canis* from the present study

Samples	Tandem repeat	Number of copies	Genogroup
Sample 20 (PZ138114)	TEDSVSAPA	16	EUA
Sample 21 (PZ138115)	TEDSVSAPA	12	EUA
Sample 26 (PZ138116)	ASVVPEAE	13	BR
Sample 38 (PZ138117)	TEDSVSAPA	13	EUA
Sample 49 (PZ138118)	TEDSVSAPA	12	EUA

**Fig. 3:**
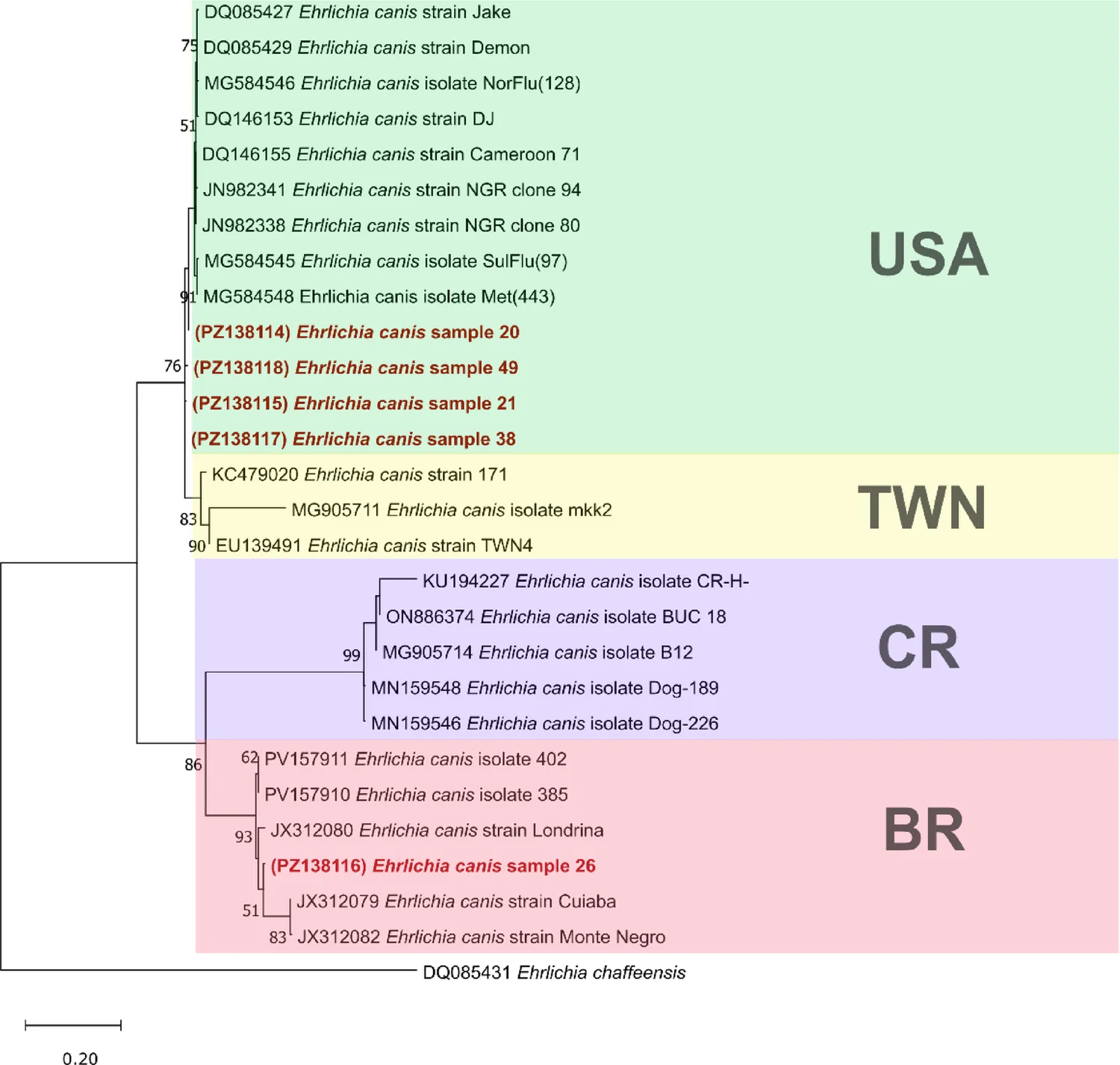
Phylogenetic tree based on a fragment of the *trp36* gene from dogs in the Unaí microregion, Minas Gerais, Brazil, constructed using the Maximum Likelihood method and the Tamura 3-parameter model. Bootstrap values are shown next to the branches. Among-site rate variation was modeled using a discrete Gamma distribution (+ G, parameter = 0.8476). The scale bar indicates the number of substitutions per site. USA, TWN, CR, and BR represent the *E. canis* genogroups

In the phylogenetic analysis of the *trp36* gene, four sequences obtained in the present study clustered with sequences from different countries belonging to the American (USA) genogroup, whereas one sequence clustered with Brazilian isolates belonging to the BR genogroup (Fig. 3). Brazilian samples, including those obtained in this study and those previously described in the literature, were distributed across different subclades.

This clustering pattern suggests the existence of genetic variation associated with the geographic distribution of isolates. The arrangement of sequences in the phylogenetic tree highlights the genetic diversity of *E. canis*, indicating the circulation of different genogroups that appear to vary according to geographic region.

## Discussion

Although canine monocytic ehrlichiosis is widely reported in Brazil, molecular data on the genetic structure of *E. canis* in Minas Gerais are still limited, particularly in naturally infected canine populations. In this context, the detection of *E. canis* in 5.4% of samples based on the *trp19* gene indicates active circulation of the agent in the Unaí microregion and contributes to the characterization of its epidemiological profile in a poorly studied area.

Differences in *E. canis* infection frequencies reported among studies are expected and likely reflect variations in study design, target populations, and diagnostic approaches [21]. Many investigations are based on convenience sampling, often including clinically suspected animals, which may overestimate prevalence compared to apparently healthy populations. In addition, ecological factors, such as vector abundance and environmental conditions, may also influence infection dynamics. Therefore, the frequency observed in the present study should be interpreted within this multifactorial context.

The molecular characterization revealed the co-circulation of Brazilian and American *E. canis* genogroups in the study area, indicating the presence of genetically distinct lineages in the same geographic region. This pattern suggests a non-homogeneous population, shaped by repeated introduction events and/or prolonged maintenance of divergent strains in an endemic environment. Genetic diversity was further evidenced by the identification of nine *p28* haplotypes, confirming high intraspecific variability. This variability may be associated with host movement, vector dynamics, and ecological connectivity among canine populations in the region [4].

Differences in amplification and variability between the *p28* and *trp36* genes may reflect distinct selective pressures acting on these loci. While *p28*, belonging to a multigene family encoding outer membrane proteins, tends to favor the detection of different strains, *trp36* shows high variability due to its tandem repeat structure and high immunogenicity. This variability has been attributed to selective pressure exerted by the host immune system, promoting antigenic diversification, immune evasion, and pathogen adaptation [22]. In addition, changes in these genes may influence the sensitivity of diagnostic assays, compromise the performance of potential vaccines [16, 23], and favor the emergence of variants with different virulence profiles [12].

Analyses of the *trp36* gene revealed two main genogroups and substantial variation in the number of tandem repeat copies, ranging from 12 to 16 repeats in the present study. Compared to other Brazilian regions and international reports, this variability reinforces the dynamic nature of this locus and its association with antigenic variation [4, 8, 24, 25]. The diversity observed in TRP36 repeats, including patterns previously described in both Brazilian and international isolates, supports the hypothesis that immune selection plays a central role in shaping *E. canis* diversity.

Despite this genetic heterogeneity, little is known about the clinical characteristics associated with each *E. canis* genogroup. Genogroups such as BR have been associated with hematological alterations, such as reduced hematocrit and hemoglobin levels, suggesting a greater impact on anemia in infected animals [26]. Different genotypes may also influence host response, affecting bone marrow regeneration, the intensity of the inflammatory response, and the clinical manifestation of infection [27]. Despite these associations, the relationship between circulating genotypes and different clinical profiles is still not fully elucidated, highlighting the need for further studies to better understand how the genetic profile of *E. canis* affects its pathogenicity and clinical outcomes in infected animals.

## Conclusion

This study demonstrated the circulation of genetically diverse *E. canis* strains in naturally infected dogs from the Unaí microregion, including the co-circulation of the Brazilian and American genogroups. These findings expand current knowledge of the molecular epidemiology of *E. canis* in Minas Gerais and highlight the value of combining multiple molecular markers to characterize the genetic diversity of circulating strains.

## Data Availability

The data supporting the findings of this study are available from the corresponding author upon reasonable request.
